# The occurrence of *Treponema* spp. in gingival plaque from dogs with varying degree of periodontal disease

**DOI:** 10.1371/journal.pone.0201888

**Published:** 2018-08-09

**Authors:** Janna Nises, Anna Rosander, Ann Pettersson, Annette Backhans

**Affiliations:** 1 Department of Clinical Sciences, Swedish University of Agricultural Sciences, Uppsala, Sweden; 2 Department of Biomedical Science and Veterinary Public Health, Swedish University of Agricultural Sciences, Uppsala, Sweden; Zhejiang University, CHINA

## Abstract

Periodontal disease is common in dogs and is initiated by gingival plaque composed of several hundred bacterial species. Some of these species have specifically been pointed out as potential periodontal pathogens, such as *Treponema* spp. *Treponema* spp. are difficult to culture and therefore the majority have been detected by culture-independent methods, such as PCR (Polymerase Chain Reaction). This leaves many *Treponema* spp. uncharacterized and unnamed. In this study, treponemes were investigated in gingival plaque from dogs with varying degree of periodontal disease with the aim to describe their occurrence and diversity in dogs. The methods used were culture, phase-contrast microscopy, PCR targeting the 16SrRNA-tRNA^Ile^ intergenic spacer region (ISR2), sequencing of the ISR2 and phylogenetic analysis. *Treponema* spp. were detected in samples from 10 out of 11 dogs and isolates were obtained from six dogs. Both healthy and periodontitis affected dogs were *Treponema* positive. Phylogenetic analysis, based on ISR2 sequences, revealed a large diversity of treponemes in the study population that were found to be distributed mainly in two groups, corresponding to the human oral treponeme phylogroups II (*Treponema denticola*) and IV (*Treponema maltophilum*) genetic groups. They were generally more distantly related to other treponemes in these groups. Treponemes from dogs with periodontitis and dogs with mild gingivitis without periodontitis did not differ in any obvious way. The results indicate that several phylotypes of oral treponemes are common in dogs regardless of periodontal status.

## Introduction

Periodontal disease, gingivitis and periodontitis, is a common affliction in dogs as well as in humans [1]. Accumulation of bacterial plaque on the tooth surface is believed to play a part in the initiation of periodontal disease, and as it develops, a shift occurs in the bacterial flora from being predominantly aerobic to predominately anaerobic [2]. Recent studies have revealed a large diversity of bacterial species in subgingival plaque from dogs, and also extensive differences between dogs and humans in the composition of species [3–6]. It was recently shown that of 353 bacterial species in dog subgingival plaque, only 16.4% were shared with humans [4]. In human patients, *Treponema denticola*, *Tannerella forsythia* and *Porphyromonas gingivalis* as a group, referred to as the “Red complex”, show a strong association to periodontitis [7–9], and some studies have indicated their involvement also in dogs [10–15]. Because of their fastidious nature [16], treponemes are difficult to cultivate and have often been excluded from culture-dependent studies [10, 17, 18]. Thus, only 10 oral treponemal species have yet been isolated and characterized: *T*. *denticola* [19], *T*. *socranskii* [20], *T*.*pectinovorum* [21], *T*.*vincentii* [22], *T*. *lecithinolyticum* [23], *T*. *amylovorum* [24], *T*. *maltophilum* [25], *T*. *medium* [26], *T*. *parvum* [27] and *T*. *putidum* [28]. So far, few studies have been published where treponemes from dogs have been cultured and isolated, and there is a limited knowledge on the occurrence and importance of these bacteria in periodontal disease in this species. Species-specific PCRs or methods like fluorescence in situ hybridization (FISH) have been used to identify treponemes from dogs at species-level, however, often, the methods used were developed to identify oral treponemes from humans [13, 29].

The aim of this study was to describe the occurrence and diversity of oral *Treponema* spp. in dogs with periodontitis by:

1. Culturing and isolation of *Treponema* spp. from plaque samples from dogs with varying degrees of periodontal disease.
2. Analysing pure spirochetal cultures and uncultured gingival plaque samples with 16S rRNA-tRNA^Ile^ intergenic spacer region PCR, using primers specific for the genus *Treponema*.
3. Sequencing of the obtained amplified PCR-products and study their genetic diversity and relation to other *Treponema* spp.

## Materials and methods

### Study population

A total of 11 dogs, of different breed, age and periodontal status, participating in a larger study on oral health and systemic diseases at the Swedish University of Agricultural Sciences, Department of Clinical Sciences, were sampled. The study was approved by the Uppsala Ethical Committee on Animal Research (Dnr: C70/15), and all owners had given their written consent prior to participating in the study. Inclusion criteria were weight >7 kg, age >1 year. The minimum body weight was set at 7 kg in order to ensure adequate body size for blood sampling. Further, as periodontitis is uncommon in animals under 1 year of age, we chose not to include animals younger than this in the study. Dogs were excluded from the study if they had received medical treatment 60 days prior to examination, had complicated tooth fractures, endodontic treatment, oral tumours or generalized gingival hyperplasia. All oral examinations were performed and recorded using standardized protocols by the same experienced veterinarian (Swedish specialist in small animal dentistry). Periodontal status was scored using the modified Total Mouth Periodontal Score-Gingivitis (TMPS-G) that has previously been validated for assessing the degree of gingival inflammation in the entire mouth [30]. TMPS-G was scored between 0 and 3, where 0 indicated overall healthy gingiva and 3 severe gingival inflammation. Clinical attachment loss (CAL) was measured in mm on the buccal, mesial, lingual and distal surface of each tooth root using a 1 mm calibrated dental probe. CAL is defined as the deepest distance from the cemento-enamel junction to bottom of the gingival pocket. No CAL can be measured in periodontally healthy teeth.

If CAL was greater than 0 periodontitis was present. Two dogs, with no signs of periodontitis and only mild signs of gingivitis (defined by pink gingiva color, less than one mm marginal hyperemia on maximum three teeth, no swelling of the gingival margin, knife-edge adaptation of gingiva to teeth, if probing could be performed only slight bleeding on probing on maximum three teeth, and no gingiva retraction) were used as controls.

### Sampling and culture

Two supragingival samples were taken from each dog and two samples were taken from periodontal pockets if present. All samples were taken on the buccal surface. One of the supragingival samples was taken with a cotton swab from the tooth surface and gingiva of several teeth, and put in a flask with liquid growth medium (see below) for isolation of *Treponema* bacteria. The other supragingival sample was taken for DNA preparation with a plastic loop and put in a tube containing 500 μl PBS (Phosphate Buffered Saline). The periodontal pocket samples were collected with a plastic loop, one was put in a 30 ml flask with liquid growth medium and the other in a tube with 500 μl PBS. Each PBS-sample was examined directly with phase-contrast microscopy and the presence of spirochetes was noted. The PBS-samples were then centrifuged in 4000 rcf for 5 minutes and the pellets were stored in -20 C until DNA extraction. These samples will further on, in this text, be referred to as “original samples”. Culturing was performed according to Svartström *et al* 2013 [31]. Shortly, flasks were prepared with 10 ml FAB + A (fastidous anaerobe broth from Lab 71 (LAB 71, Lab M, Heywood, Lancashire, UK) prepared with 2 grams of d-glucose per liter) with 720 μg thiamine pyrophosphate per liter, 10 μl each of isobutyric acid, isovaleric acid, 2-methylbutyric acid, valeric acid, solubilized in 0.1 M potassium hydroxide, 25% fetal calf serum, S 0115, (Biochrom AG, Germany), 10 μg/ml rifampicin Sigma (Sigma–Aldrich Sweden AB), and 10 μg/ml enrofloxacin (Fluka, Sigma–Aldrich Sweden AB)) and were incubated anaerobically at 37°C and 90 rpm. Cultures were monitored macroscopically and by phase contrast microscope about once a week for detection of spirochetal growth. From cultures with spirochete growth, 100 μl was added on a filter (pore size 0.22 μm) placed directly on a fastidious anaerobe agar plate; (FAA plate, National Veterinary Institute, Uppsala, Sweden). The filter was removed after 2–3 days. Once bacterial growth was detected a piece of agar was transferred to FAB + A with supplements but without antibiotics. Cultures with spirochetes in abundance (about 10 spirochetes/field of vision), and free from other bacteria were frozen with glycerol in -80°C and a bacterial pellet was stored in -20°C for later DNA extraction.

### Molecular analysis

For DNA extraction, pellets from isolates were washed once in PBS whereas pellets from original samples were not. Pellets were mixed with 50 μl Super-Q water, boiled for 10 min and centrifuged in 16200 rcf for 2 min at 4°C, the supernatants were stored in -20°C. PCR amplification of the 16SrRNA-tRNA^Ile^ intergenic spacer region (ISR2) was performed using primers selective for the genus *Treponema* and the protocol described by Stamm *et al*. [32]. Agarose gel electrophoresis was performed to verify successful amplification, and if no or very weak bands were obtained, samples were run again with 35 PCR cycles. The PCR-products were sent for sequencing at Macrogen Inc, Korea using PCR primers as sequencing primers. Sequencing results were confirmed by sequencing of multiple PCR-products from the same sample. PCR-products from all original samples and from isolates with sequences showing signs of mixed signals, were first cloned using the TOPO TA Cloning Kit for Sequencing, pCR 4-TOPO Vector (Invitrogen by Life Technologies). Transformation into competent *Escherichia coli* (One Shot TOP10 chemically competent cells) was made using the Rapid One Shot chemical transformation protocol; 50 μl of the cells were then spread on prewarmed LB (Luria-Bertani)-agar plates containing 50 μg/ml ampicillin, and incubated for 24 hours at 37°C. Four isolated colonies from each plate were cultured in tubes with 5 ml LB broth and 5 μl ampicillin (50 μg/ml final concentration) at 37°C and 160 rpm for 24 hours. The cultures were pelleted by centrifugation at 2900 rcf for 15 minutes, and plasmids were purified using the GeneJET Plasmid Miniprep Kit (Fermentas Life sciences) and eluted with BPC grade water. The plasmids were sent for sequencing at Macrogen Inc, Korea, using general primer M13F. Sequences were trimmed in CLC Main Workbench 7.8 (CLC bio). Homology searches were performed using the Basic Local Alignment Search Tool (BLAST) algorithm at National Center for Biotechnology Information (NCBI) [33]. Progressive alignments of sequences obtained from the study and sequences with high identity from the homology searches were made with default gap cost parameters and the “very accurate (slow)” algorithm and a phylogenetic tree was constructed. The “Model testing” tool in CLC Main Workbench 7.8 (CLC bio) showed that the best fit model was General Time Reversible (GTR), that was used to construct a maximum likelihood phylogenetic tree. If identical sequences were derived from the same sample only one was included in the tree. For isolates considered “pure” based on ISR-sequences, the 16S rRNA gene was amplified and sequenced using the primer pair pA and pH [34]. A phylogenetic tree was constructed with parts of the 16S rRNA including representatives of the human oral treponeme phylogroups. Accessions numbers of all GenBank derived sequences can be found in S2 Table.

## Results

### Study population

Seven male and four female dogs of different breeds were sampled. Their age varied between 3–12 years (mean 7.9 years, SD 2.9) and weight 7–46 kg (mean 20.6 kg, SD 12.6). The TMPS-G scores and CAL at the sampling sites are shown in Table 1. Supragingival samples were collected from all 11 dogs and additional samples from periodontal pockets were collected from seven of the dogs. Tooth pocket depth in dogs with periodontitis ranged from 4–10 mm.

**Table 1:** Periodontal status of the dogs, findings of *Treponema* spp. in individual dog samples, and PCR-positive original sample and isolate names.

Dog	TMPS-G	CAL (mm)	Spirochetes visible by microscopy		Isolates	PCR-positive samples	Sample site	Isolate	Sample site
			Supra gingival	Periodontal pocket					
1	1.92	4	Yes, later in culture	Yes, directly after sampling	Yes	H1E2	Periodontal pocket	THI1b	Periodontal pocket
2	1.69	0	Yes, later in culture	n.t	Yes	None	-	THI5	Supragingival surface
3	2.46	4	Yes, directly after sampling	Yes, directly after sampling	Yes	H3E1	Periodontal pocket	THI2	Periodontal pocket
								THI2b	Periodontal pocket
4	1.85	4	No	No	No	None	-	No	
5	1.93	5	No	Yes, directly after sampling	No	H5E1	Periodontal pocket	No	-
						H5E2	Supragingival surface	No	-
6	2.65	n.t	Yes, directly after sampling	n.t	No	H6E1	Supragingival surface	No	-
7	2.87	5	Yes, directly after sampling	Yes, directly after sampling	Yes	H7E2	Periodontal pocket	THI4a	Supragingival surface
								THI4b	Periodontal pocket
8	1.87	4	No	Yes, directly after sampling	Yes	H8E1	Supragingival surface	THI6	Periodontal pocket
						H8E2	Periodontal pocket	THI6b	Periodontal pocket
9*	n.t	n.t	Yes, directly after sampling	n.t	Yes	H9E1	Supragingival surface	THI7	Supragingival surface
10	2.18	10	No	Yes, directly after sampling	No	H10E2	Periodontal pocket	No	-
11*	1.20	0	Yes, directly after sampling	n.t	No	H11E1	Supragingival surface	No	-

TMPS-G = Total Mouth Periodontal Score gingival) was scored between 0–3, where 0 is healthy gingiva and 3 is severe gingival inflammation. [21], CAL = Clinical attachment loss, n.t = not tested

* = “control dogs”, i.e. dogs with mild gingivitis without periodontitis.

### Sampling and culture

Spirochetes were observed by phase contrast microscopy in original or culture samples from 10 of the dogs, and were more commonly seen in samples from tooth pockets (6/7, 86%) than from supragingival samples (7/11, 64%). Results are presented in Table 1. Spirochetes were seen in the supragingival samples of both control dogs. Spirochetes were easily seen in samples from periodontitis affected dogs, two to four in the first field of vision, while several fields of vision had to be examined before spotting even one spirochete in other samples. A total of nine isolates of spirochetes (pure from non-spirochaetal bacteria as determined by phase-contrast microscopy) were obtained from six different dogs. Isolates were obtained from both supragingival samples and from periodontal pocket samples as well as from dogs with periodontitis and no periodontitis, including one of the control dogs (Table 1). Incubation time to obtain a pure culture of spirochetes with a density of at least 10 spirochetes/field of vision was between one and a half month and five months. From some of the cultures that initially contained large amounts of spirochetes, it was not possible to obtain a pure culture whereas some cultures, with no initial visible spirochetal growth, were positive after several months.

### Molecular analysis

All isolates and 12 of 18 original samples were positive by PCR. Of the supragingival samples, five of 11 were positive by PCR. Six of seven samples from periodontal pockets were positive by PCR. The negative PCR sample was also negative in phase contrast microscopy.

#### ISR2 sequences

In the original samples, different *Treponema* spp. may be present, which could generate similar size PCR fragments. For this reason, original sample PCR products were cloned into the pCR 4-TOPO Vector and four clones from each sample were chosen for sequencing to enhance the possibility of detecting different treponemes. The same approach was used on PCR products from isolates for which a first round of sequencing showed double peaks, indicating a mixed population. Twenty ISR2 sequences were obtained from isolates, of which 15 were deposited in GenBank (accession numbers MH482781-95). From four isolates complete ISR2 sequences (GenBank accession numbers MH482746-49) were assembled which were considered “pure” after up to five amplicons from the same sample yielded identical sequences. From original samples 38 sequences were retrieved and 31 were deposited in GenBank (accession numbers MH482750-80). Names of the sequences and accession numbers can be found in S1 Table. The most closely related sequences (highest identity score on NCBI-blast) for isolates and original samples are listed in Tables 2 and 3, respectively. Only two of the closely related sequences originated from isolates: *Treponema denticola* ATCC 35405 and *Treponema* sp. isoM1187, the rest were from uncultured clones. Four of the sequences originated from bovine ulcerative mammary dermatitis, three from porcine gingiva, and remaining sequences came from porcine ear necrosis and porcine shoulder ulcer.

**Table 2:** Designation of obtained isolates and BLAST homology of isolate ISR2 sequences.

Dog	Isolate	ISR2 length (nucleotides)	Closest related sequence (BLAST homology)	Accession number (GenBank)	Identity (%)	Query cover (%)
1	THI1b Clone B	315	Uncultured Treponema sp. clone E1163b	KC494458	98	66
	THI1b Clone C	315	Uncultured Treponema sp. clone E1163b	KC494458	98	66
	THI1b Clone D	315	Uncultured *Treponema* sp. clone E1163b	KC494458	99	66
2	THI5	322	*Treponema* sp. isoM1187	KC619321	86	100
3	THI2 Clone A	316	Uncultured *Treponema* sp. clone C1BT2-8	AY342046	87	100
	THI2 Clone B	282	*Treponema denticola* ATCC 35405	AE017226	94	100
	THI2 Clone C	318	Uncultured Treponema sp. clone E1163b	KC494458	99	65
	THI2 Clone D	314	Uncultured *Treponema* sp. clone C1BT2-8	AY342046	87	100
	THI2b	314	Uncultured *Treponema* sp. clone C1BT2-8	AY342046	87	100
7	THI4a	322	*Treponema* sp. isoM1187	KC619321	86	100
	THI4b	322	*Treponema* sp. isoM1187	KC619321	86	100
8	THI6 Clone A	322	Treponema sp. isoM1187	KC619321	86	100
	THI6 Clone B	322	Treponema sp. isoM1187	KC619321	86	100
	THI6 Clone C	315	Uncultured Treponema sp. clone C1BT2-8	AY342046	87	100
	THI6 Clone D	315	Uncultured Treponema sp. clone C1BT2-8	AY342046	87	100
	THI6b Clone A	315	Uncultured Treponema sp. clone C1BT2-8	AY342046	87	100
	THI6b Clone B	315	Uncultured Treponema sp. clone C1BT2-8	AY342046	87	100
	THI6b Clone C	315	Uncultured Treponema sp. clone E1163b	KC494458	99	66
	THI6b Clone D	315	Uncultured Treponema sp. clone C1BT2-8	AY342046	87	100
9	THI7	314	Uncultured Treponema sp. clone C1BT2-8	AY342046	88	100

**Table 3:** Designation of original samples and BLAST homology of ISR2 sequences from original sample clones.

**Dog**	**Sequence from** **original samples**	**ISR2 length (nucleotides)**	**Closest related sequence (BLAST homology)**	**Identity** **(%)**	**Query cover (%)**
1	H1E2 Clone A	313	*Treponema* sp. isoM1187	86	100
	H1E2 Clone B	282	*Treponema denticola* ATCC 35405	92	100
	H1E2 Clone D	284	*Treponema denticola* ATCC 35405	94	100
3	H3E1 Clone A	282	*Treponema denticola* ATCC 35405	95	100
	H3E1 Clone B	282	*Treponema denticola* ATCC 35405	94	100
	H3E1 Clone C	292	*Treponema putidum* strain OMZ 758	96	47
	H3E1 Clone D	265	*Treponema denticola* ATCC 35405	90	100
5	H5E1 Clone A	265	*Treponema denticola* ATCC 35405	91	100
	H5E1 Clone B	312	*Treponema* sp. isoM1187	86	100
	H5E1 Clone C	265	*Treponema denticola* ATCC 35405	91	100
	H5E1 Clone D	280	Uncult. *Treponema* sp. clone C1BF-3	88	100
	H5E2 Clone A	318	Uncult. *Treponema* sp. clone E1163b	97	66
	H5E2 Clone B	265	*Treponema denticola* ATCC 35405	91	100
	H5E2 Clone C	265	*Treponema denticola* ATCC 35405	91	100
	H5E2 Clone D	313	Uncult. *Treponema* sp. clone C1BT2-8	88	100
6	H6E1 Clone A	265	*Treponema denticola* ATCC 35405	90	100
	H6E1 Clone B	265	*Treponema denticola* ATCC 35405	88	100
	H6E1 Clone C	281	*Treponema denticola* ATCC 35405	94	100
7	H7E2 Clone A	284	*Treponema denticola* ATCC 35405	94	100
	H7E2 Clone B	282	*Treponema denticola* ATCC 35405	93	93
	H7E2 Clone C	282	*Treponema denticola* ATCC 35405	94	100
	H7E2 Clone D	245	*Treponema* sp. C1UD2	96	100
8	H8E1 Clone C	245	*Treponema pedis* strain isoM1224	98	85
	H8E1 Clone D	318	*Uncultured Treponema* sp. clone E1163b	95	66
	H8E2 Clone A	244	*Treponema denticola* ATCC 35405	93	59
	H8E2 Clone B	273	*Treponema putidum* strain OMZ 758	97	52
	H8E2 Clone C	265	*Treponema denticola* ATCC 35405	90	100
	H8E2 Clone D	273	*Treponema putidum* strain OMZ 758	97	52
9	H9E1 Clone A	317	Uncult. *Treponema* sp. clone C1 BT2-8	87	100
	H9E1 Clone B	259	*Treponema parvum* strain isoB1119	88	100
	H9E1 Clone C	246	Uncult. *Treponema* sp. clone C2BT2-8	90	100
	H9E1 Clone D	282	*Treponema denticola* ATCC 35405	94	100
10	H10E2 Clone A	282	*Treponema denticola* ATCC 35405	94	100
	H10E2 Clone B	244	*Treponema pedis* strain isoM1224	98	85
11	H11E1 Clone A	266	*Treponema denticola* ATCC 35405	88	100
	H11E1 Clone B	313	Uncult. *Treponema* sp. clone C1BT2-8	89	100
	H11E1 Clone C	282	*Treponema denticola* ATCC 35405	94	100
	H11E1 Clone D	313	Uncult. *Treponema* sp. clone C1BT2-8	88	100

#### Analysis of genetic diversity

**Fig 1:**
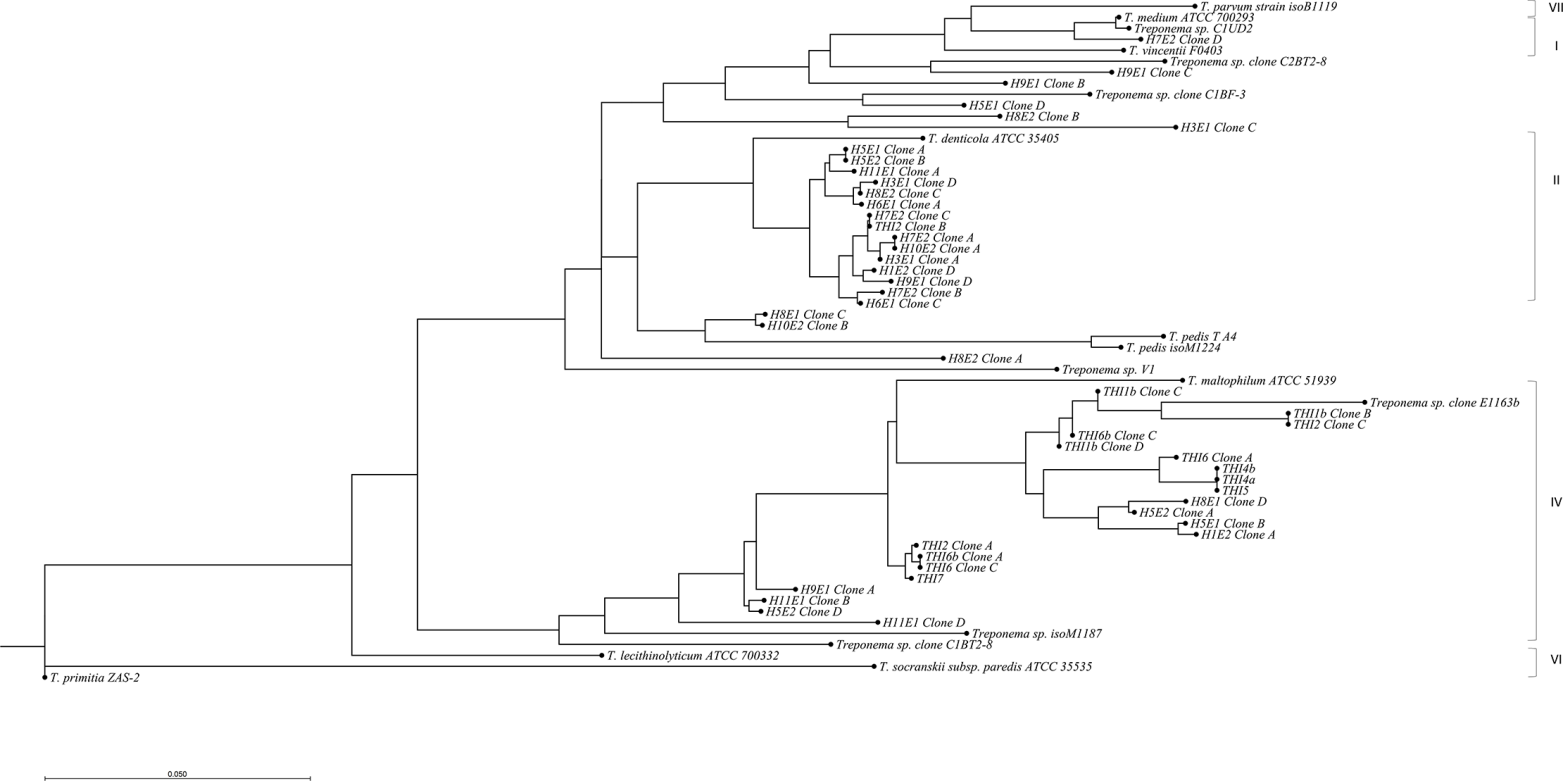
Phylogenetic relationship based on distance matrix analysis of the 16SrRNA-tRNA^Ile^ intergenic spacer region (ISR2). Sequences included: *Treponema* spp. from dogs in this study and sequences from GenBank representing the phylogroups for human oral treponemes based on 16S rRNA sequences proposed by Dewhirst et al (2010). Accession numbers and origin for all the sequences from this study are found in S1 Table.

Fig 1 shows the phylogenetic tree constructed by the *Treponema* spp. derived from dogs in this study, closest related sequences by BLAST, and representatives from the different phylogroups in the systematic classification of human oral treponemes proposed by Dewhirst *et al*. [35]. Accession numbers and origin for all the sequences are found in S1 and S2 Tables. The sequences from dogs appeared in phylogroups I, II and IV, but also clustered with *T*. *pedis*. The majority of ISR2 sequences appeared in two main groups, of which one consisted of the phylogroup II (*T*. *denticola)*, *Treponema* sp. clone C1BF-3-related sequences and *T*. *pedis*-related sequences. The other group clustered with phylogroup IV in two main clusters, one that comprised *T*. *maltophilum* and the other one comprised clone E1163b, originating from porcine ear necrosis. The remaining sequences clustered with *Treponema medium* (phylogroup I) and *T*. *parvum*. All obtained isolates grouped with the phylogroup IV associated group except one that was in phylogroup II clustering with *T*. *denticola*. In all samples but from one dog, more than one treponemal sequence was derived. The sequences from the two control dogs were found in both main groups.

**Fig 2:**
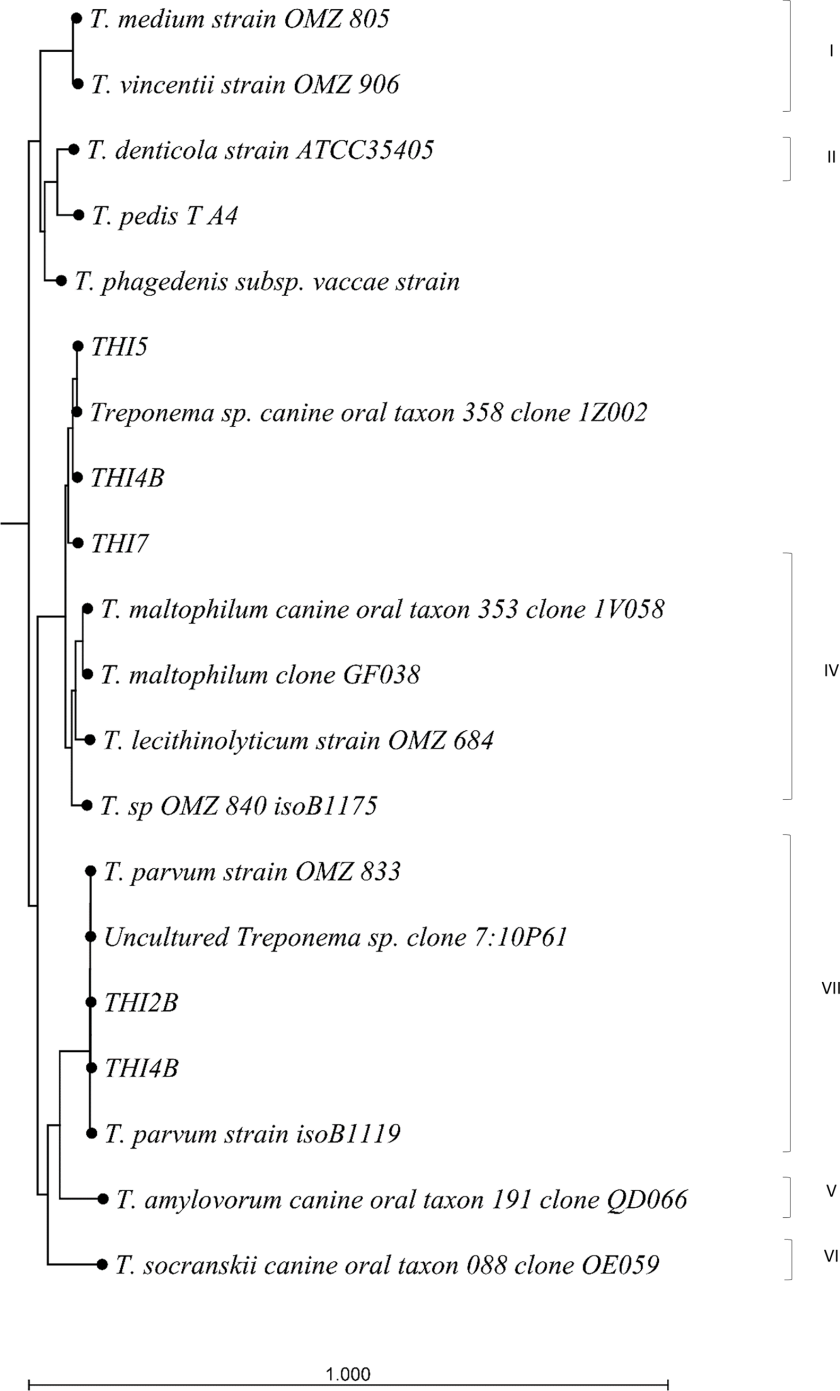
Phylogenetic relationship based on distance matrix analysis of 16S rRNA sequences from *Treponema* spp. from isolates obtained in this study and sequences from phylogroups 1–7 derived from GenBank.

Sequencing of the 16S rRNA resulted in about 1115 basepair long sequences of good quality. Two of the isolates, THI4a and b, that had identical ISR2 sequences, both showed double peaks on the chromatogram and was dismissed for further phylogenetic analysis. The 16S rRNA gene phylogenetic tree (Fig 2) showed that the isolates clustered with *T*. *parvum* or *Treponema* sp. canine oral taxon 356 clone and *T*. *maltophilum*.

### Data analysis

The distribution of supragingival and periodontal treponemes in the phylogenetic groups and clusters are shown in Table 4.

**Table 4:** Background data of the phylogenetic groups and clusters.

Cluster	Description	Proportion of supragingival surface derived Treponemes (%)	Proportion of periodontal pocket derived Treponemes (%)
II	Phylogroup II, *Treponema denticola*	5/17 (30)	9/27 (33)
II b	*Treponema* sp. clone C1BF-3	4/17 (24)	1/27 (27)
II c	*Treponema pedis*	1/17 (6)	1/27 (4)
**SUM Group II**		10/17 (59)	11/27 (41)
IV a	*Treponema maltophilum*	3/17 (18)	4/27 (15)
IV b	*Treponema* sp. clone E1163b	4/17 (24)	7/27 (26)
**SUM Group IV**		7/17 (41)	22/27 (66)
Other		3/17 (18)	7/27 (26)

As shown in Table 1, a larger proportion of periodontal pocket-derived treponemes clustered with phylogroup IV than with phylogroup II, whereas more supragingival-derived treponemes clustered with phylogroup IV.

## Discussion

The role of *Treponema* spp. in the etiology of periodontal disease is unclear. Several studies have related their presence in gingival plaque to the development of periodontal disease in humans [7, 9, 34] and also in dogs [13, 14]. However little is known of what treponemal species are of importance in dogs, and isolation has only been performed in one previous study [29]. The PCR results and the results from the microscopy, we detected treponema in supragingival plaque and/or periodontal pockets from 10 out of 11 dogs, and isolates were obtained from six of the dogs.

An indication of the relative amount of spirochetes in the samples was given by observation of spirochetes using phase-contrast microscopy directly after sampling or during culturing. Spirochetes were easier to detect by phase-contrast microscopy in the tooth-pocket samples. Notably, spirochetes were detected by phase-contrast microscopy directly after sampling in both dogs with no periodontitis or mild gingivitis, which suggests that spirochetes are common in the oral microflora. The PCR results and the results from the microscopy directly after sampling correlated well for the periodontal pocket samples, whereas for the supragingival samples, five were positive by microscopy of which two were negative by PCR. From two of these samples isolates were obtained after a longer time period of culture. Possible explanations are that the quantity of bacteria in some samples was below the detection limit for the PCR, or that inhibitory factors were present in the samples. The primer pair used may also not fit with all oral spirochetes.

Together, the results indicate that the periodontal pocket samples contained more *Treponema* than the supragingival samples, an expected result as pockets creates a more anaerobic environment and a hideaway place for bacteria, and it is also in agreement with several other studies [13, 14]. Clearly a swipe with a plastic loop in a periodontal pocket generated more material than a swipe along the supragingival surface. Thus, for future studies, a method for supragingival sampling should be used which result in more material.

Two of the three supragingival isolates came from dogs without periodontitis. One dog was used as a control (mild gingivitis), and in the sample from this dog three spirochetes were observed in the first field of vision directly after sampling, which was similar to what was seen in subgingival samples from periodontitis affected dogs. It was also interesting that the only dog negative for *Treponema*, by PCR and also by microscopy, had periodontitis. This shows that, by these methods, *Treponema* spp. can appear quite abundant in more or less periodontally healthy dogs and uncommon in periodontitis affected dogs, even if the opposite seems to be more common. Several previous studies have also found oral *Treponema* in both healthy and periodontitis affected dogs [3, 6, 13]. Nordhoff and co-workers [13], found *Treponema* spp. more often with more severe periodontal disease, whereas Davis [3] and Wallis [6] found a reduction in health associated bacterial species to be more correlated with periodontal disease than an increase of putative periodontal pathogens such as *Treponema* spp. Thus, the significance of *Treponema* spp. in the etiology of periodontitis is still unclear and needs further investigation.

Culturing *Treponema* spp. is very time consuming and the possibility of obtaining a pure isolate seem unrelated to the amount of spirochetes in the original culture. As a result, not many strains have been described, especially not from dogs, compared to more easily grown bacteria. The methods used in the present study were those described previously by Svartström *et al*., [31], but for some of the treponemes, the methods could be further optimized. One observation made was that cultures regarded as “hopeless”, after being left for several weeks in an unopened anaerobic jar, demonstrated dense population of apparently healthy spirochetes. One isolate was obtained first after five months. This indicate that some strains of *Treponema* spp. require a substantially longer incubation time.

One aim of our study was to analyse the diversity of oral *Treponema* in dogs. We chose to do this by comparing sequences of the 16SrRNA-tRNA^Ile^ intergenic spacer region because it is believed to differentiate better between closely related *Treponema* spp. than 16S rRNA analysis [32]. However a drawback with the ISR2 region is the relatively few deposited *Treponema* spp. sequences in Genbank, that makes comparison to for example human oral treponemes difficult. Another problem is that some *Treponema* spp. will not be detected by the ISR2 PCR. Thus, considering the limitations of our method, the *Treponema* spp. oral flora is likely to be more diverse, as has been implicated in recent studies [36]. However, our result show that dogs harbour treponemes similar to phylogroups I, II, IV and also *T*. *pedis*, which is in accordance with the study by Nordhoff *et al*. (2008) where *Treponema* spp. from phylogenetic groups I, II, IV and VI was found in subgingival plaque samples from dogs using *Treponema* specific oligonucleotide probes [13]. Similarly, Valdez *et al* (2000) identified *T*. *denticola*, *T*. *socranskii*, *T*. *vincentii*, *T*. *maltophilum*, *T*. *medium*, *T*. *pectinovorum* by species-specific PCRs [29]. Our results also show that individual dogs are colonized by several different phylotypes of oral *Treponema* spp. In our study there was a tendency to more *Treponema* spp. from supragingival plaque clustering in group II and *Treponema* spp. from periodontal pockets in group IV. We also did not see that sequences clustered depending on the periodontal status of the dogs. However, the isolates were concentrated to one group in the phylogenetic tree, corresponding to the phylogroup IV, which indicates that either these *Treponema* spp. were easier to culture, or occurred in larger numbers in our sample material. Limitations of the present study to consider are the relatively small number of dogs, and the lack of completely healthy control dogs. However, the control dogs in this study had extremely mild gingivitis without any signs of attachment loss and that is generally considered normal within an adult dog population.

## Conclusion

Dogs harbour several different *Treponema* spp. in their oral cavity and they can be common in both healthy and periodontitis affected dogs, indicating they are part of the normal bacterial flora. A comparison of the ISR2 gene distributed the majority of these treponemes in two main groups that could be further divided in several more or less distinct clusters, related to human oral treponeme phylogroups I, II, IV. All but one of the cultivable treponemes clustered with the group IV. The random distribution of the sequences from both periodontitis free and periodontitis affected dogs does not indicate that a specific phylotype is more involved in the etiology of periodontitis in dogs.

## Supporting information

S1 Table Genbank accession numbers for ISR2 sequences of *Treponema* spp. derived from sampling of dog gingiva and periodontal pockets. (DOCX)

S2 Table Accession numbers of sequences derived from GenBank used in this study. (DOCX)

S1 File Pairwise comparison of ISR2 sequences, using the parameter “percent identity” in CLC Main Workbench 7.9.1. (TIF)
