# Supplementary material for: The occurrence of Treponema spp. in gingival plaque from dogs with varying degree of periodontal disease
Source: PLoS One. 2018 Aug 9;13(8):e0201888. doi: 10.1371/journal.pone.0201888 (PMC6084996; doi:10.1371/journal.pone.0201888)
Supplement: S1 Table — (DOCX) [file pone.0201888.s001.docx]

**Supporting information**

**S1 Table.** Genbank accession numbers for ISR2 sequences of *Treponema* spp. derived from sampling of dog gingiva and periodontal pockets.

| Sequence ID | Accession number GenBank |
| --- | --- |
| THI5 | MH482746 |
| THI7 | MH482747 |
| THI4a | MH482748 |
| THI4b | MH482749 |
|  |  |
| THI1b_Clone_B | MH482781 |
| THI1b_Clone_C | MH482782 |
| THI1b_Clone_D | MH482783 |
| THI2_Clone_A | MH482784 |
| THI2_Clone_B | MH482785 |
| THI2_Clone_C | MH482786 |
| THI2_Clone_D | MH482787 |
| THI6_Clone_A | MH482788 |
| THI6_Clone_B | MH482789 |
| THI6_Clone_C | MH482790 |
| THI6_Clone_D | MH482791 |
| THI6b_Clone_A | MH482792 |
| THI6b_Clone_B | MH482793 |
| THI6b_Clone_C | MH482794 |
| THI6b_Clone_D | MH482795 |
|  |  |
| H1E2_Clone_A | MH482750 |
| H1E2_Clone_D | MH482751 |
| H3E1_Clone_A | MH482752 |
| H3E1_Clone_C | MH482753 |
| H3E1_Clone_D | MH482754 |
| H5E1_Clone_A | MH482755 |
| H5E1_Clone_B | MH482756 |
| H5E1_Clone_D | MH482757 |
| H5E2_Clone_A | MH482758 |
| H5E2_Clone_B | MH482759 |
| H5E2_Clone_D | MH482760 |
| H6E1_Clone_A | MH482761 |
| H6E1_Clone_C | MH482762 |
| H7E2_Clone_A | MH482763 |
| H7E2_Clone_B | MH482764 |
| H7E2_Clone_C | MH482765 |
| H7E2_Clone_D | MH482766 |
| H8E2_Clone_A | MH482767 |
| H8E2_Clone_B | MH482768 |
| H8E2_Clone_C | MH482769 |
| H8E1_Clone_C | MH482770 |
| H8E1_Clone_D | MH482771 |
| H9E1_Clone_A | MH482772 |
| H9E1_Clone_B | MH482773 |
| H9E1_Clone_C | MH482774 |
| H9E1_Clone_D | MH482775 |
| H10E2_Clone_A | MH482776 |
| H10E2_Clone_B | MH482777 |
| H11E1_Clone_A | MH482778 |
| H11E1_Clone_B | MH482779 |
| H11E1_Clone_D | MH482780 |
