# Supplementary material for: The occurrence of Treponema spp. in gingival plaque from dogs with varying degree of periodontal disease
Source: PLoS One. 2018 Aug 9;13(8):e0201888. doi: 10.1371/journal.pone.0201888 (PMC6084996; doi:10.1371/journal.pone.0201888)
Supplement: S2 Table — (DOCX) [file pone.0201888.s002.docx]

**Supporting information**

**S2 Table.** Accession numbers of sequences derived from GenBank used in this study.

| Sequence | Accession number (GenBank) | Sequence used in this article | Sample origin |
| --- | --- | --- | --- |
| Treponema denticola ATCC 35405 | AE017226 | 16S-23S ribosomal DNA intergenic spacer region | Human |
| Treponema sp. isoM1187 | KC619321 | 16S-23S ribosomal DNA intergenic spacer region | Pig gingiva |
| Uncultured Treponema sp. clone C1BT2-8 | AY342046 | 16S-23S ribosomal DNA intergenic spacer region | Bovine ulcerative mammary dermatitis lesion |
| Uncultured Treponema sp. clone E1163b | KC494458 | 16S-23S ribosomal DNA intergenic spacer region | Porcine ear necrosis |
| Uncultured Treponema sp. clone C2BT2-8 | AY342050 | 16S-23S ribosomal DNA intergenic spacer region | Bovine ulcerative mammary dermatitis lesion |
| Treponema pedis strain isoM1224 | KC619317 | 16S-23S ribosomal DNA intergenic spacer region | Pig gingiva |
| Treponema sp. V1 | EU410484 | 16S-23S ribosomal DNA intergenic spacer region | Cattle |
| Treponema primitia ZAS-2 | CP001843 | 16S-23S ribosomal DNA intergenic spacer region | Termite gut |
| Treponema maltophilum ATCC 51939 | NZ_KE332518 | 16S-23S ribosomal DNA intergenic spacer region | Human periodontal lesion |
| Treponema lecithinolyticum ATCC 700332 | NZ_KI260562 | 16S-23S ribosomal DNA intergenic spacer region | Oral cavity- mouth |
| Treponema vincentii F0403 | NZ_KE332514 | 16S-23S ribosomal DNA intergenic spacer region | Human periodont |
| Treponema socranskii subsp. paredis ATCC 35535 | NZ_KE332515 | 16S-23S ribosomal DNA intergenic spacer region | Human |
| Treponema medium ATCC 700293 | ATFE01000011 | 16S-23S ribosomal DNA intergenic spacer region | Human |
| Treponema parvum strain isoB1119 | KC619318 | 16S-23S ribosomal DNA intergenic spacer region | Porcine shoulder ulcer |
| Treponema pedis strain T A4 | KC619310 | 16S-23S ribosomal DNA intergenic spacer region | Porcine ear necrosis |
| Treponema sp. clone C1BF-3 | AY342051 | 16S-23S ribosomal DNA intergenic spacer region | Bovine ulcerative mammary dermatitis lesion |
| Treponema sp. C1UD2 | AY342042 | 16S-23S ribosomal DNA intergenic spacer region | Bovine ulcerative mammary dermatitis lesion |
| Treponema medium strain OMZ 805 | KT192148 | 16S rRNA |  |
| Treponema vincentii strain OMZ 906 | KT192159 | 16S rRNA |  |
| Treponema denticola strain ATCC35405 | AF139203.1 | 16S rRNA |  |
| Treponema pedis T A4 | FJ805836 | 16S rRNA |  |
| Treponema phagedenis subsp. vaccae strain | KJ206529 | 16S rRNA | Foot lesion goat |
| Treponema sp. canine oral taxon 358 clone 1Z002 | JN713528 | 16S rRNA | oral cavity subgingival plaque dog |
| Treponema maltophilum canine oral taxon 353 clone 1V058 | JN713522 | 16S rRNA | oral cavity subgingival plaque dog |
| Treponema maltophilum clone GF038 | GU420697.1 | 16S rRNA | oral cavity homo sapiens |
| Treponema lecithinolyticum strain OMZ 684 | NR_026247 | 16S rRNA |  |
| T. sp OMZ 840 isoB1175 | KC619324 | 16S rRNA | shoulder ulcer pig |
| Treponema parvum ATCC 700770 strain OMZ 833 | NR_025131 | 16S rRNA |  |
| Uncultured Treponema sp. clone 7:10P61 | JQ654137 | 16S rRNA | oral cavity subgingival plaque homo sapiens |
| Treponema parvum strain isoB1119 | KC619323 | 16S rRNA | Pig gingiva |
| Treponema amylovorum canine oral taxon 191 clone QD066 | JN713358 | 16S rRNA | Dog oral cavity subgingival plaque |
| Treponema socranskii canine oral taxon 088 clone OE059 | JN713251 | 16S rRNA | Dog oral cavity subgingival plaque |
