# Supplementary figures and images for: The occurrence of Treponema spp. in gingival plaque from dogs with varying degree of periodontal disease
Source: PLoS One. 2018 Aug 9;13(8):e0201888. doi: 10.1371/journal.pone.0201888 (PMC6084996; doi:10.1371/journal.pone.0201888)

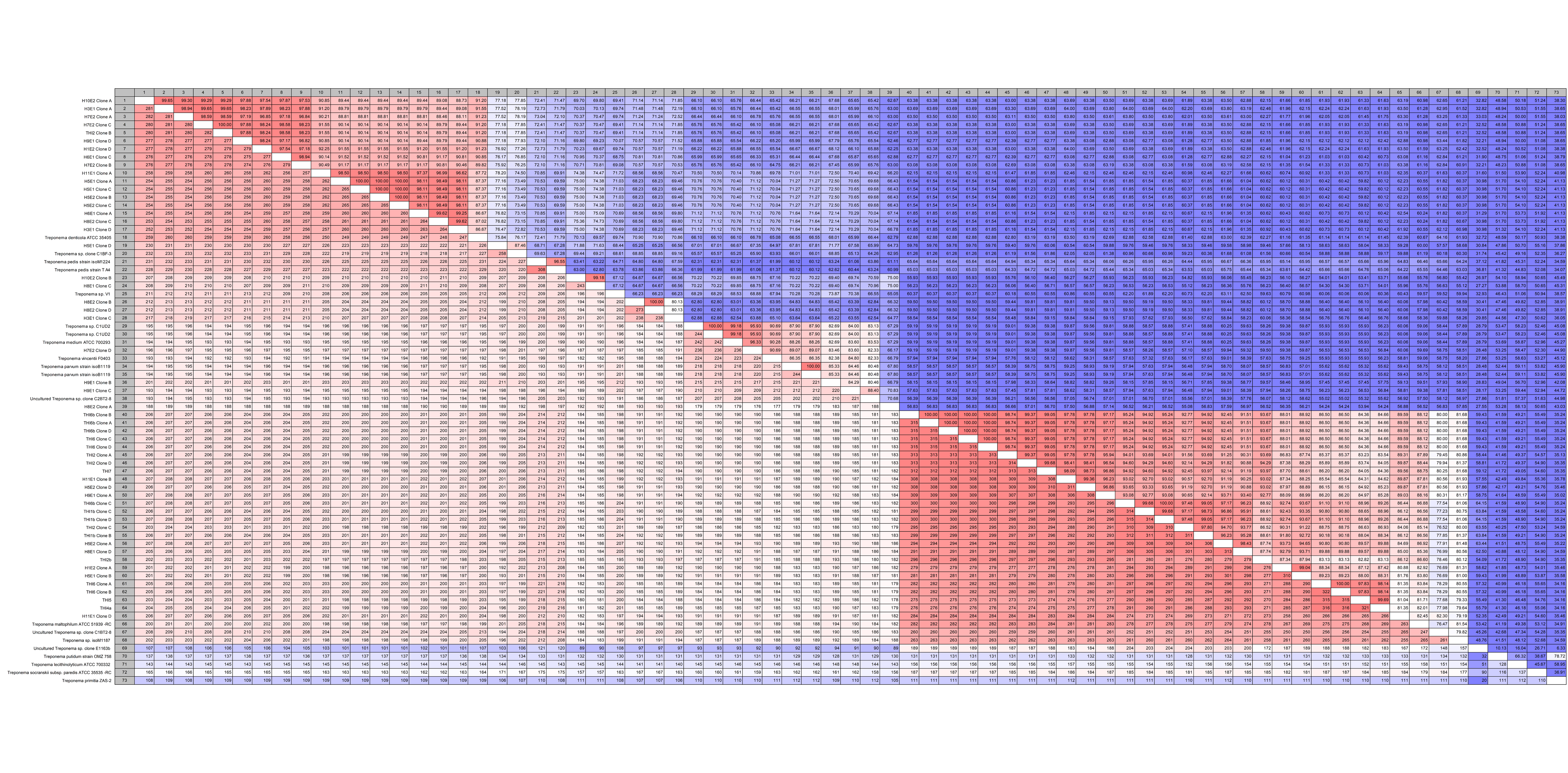

Supplement: S1 File — (TIF) [file pone.0201888.s003.tif]
